# CA 125 regression after two completed cycles of chemotherapy: lack of prediction for long-term survival in patients with advanced ovarian cancer

**DOI:** 10.1038/sj.bjc.6690744

**Published:** 1999-10

**Authors:** C Peters-Engl, A Obermair, H Heinzl, P Buxbaum, P Sevelda, M Medl

**Affiliations:** Department of Obstetrics and Gynecology, Lainz Medical Center, Wolkersbergenstraße 1, Vienna, A-1130, Austria; Departments of Gynecology and Obstetrics, Medical Computer Sciences/Clinical Biometrics, University Hospital of Vienna, Vienna, Austria

**Keywords:** ovarian cancer, CA 125, prognosis

## Abstract

The prognostic influence of CA 125 regression between the time point before surgery and after two completed courses of chemotherapy was studied in 210 patients with advanced ovarian cancer, and was compared to other well established prognostic factors. CA 125 blood samples were collected preoperatively (CA 125 pre) and 3 months after surgery (CA 125 3 mo) (at the beginning of the 3rd cycle of chemotherapy). The parameter CA 125 regression defined as log_10_ (CA 125 3 mo/CA 125 pre) was used for statistical analysis. In a survival analysis using a Cox proportional hazards model, CA 125 regression (*P* = 0.0001), residual tumour (*P* = 0.0001), age (*P* = 0.0095) and grading (*P* = 0.044) were independent variables, whereas stage of disease, histology, ascites and type of surgery failed to retain significance. Using log_10_ (CA 125 3 mo/CA 125 pre) as simple covariate in a Cox model showed a hazard ratio of 1.70 (95% confidence interval 1.32–2.19, *P* = 0.0001). However, a detailed analysis of the interaction of time with the prognostic factor CA 125 regression on survival revealed a strong time-dependent effect with a hazard ratio of more than 6 immediately after two courses of chemotherapy, whereas within approximately 1 year the hazard ratio for the surviving patients dropped quickly to the neutral level of 1. In summary, CA 125 regression is an independent prognostic factor for survival of women with advanced ovarian cancer and allows an identification of a high-risk population among patients with advanced ovarian cancer. However, the discriminating power of serial CA 125 for long-term survival seems to be temporary and prediction of individual patients outcome is far less precise. © 1999 Cancer Research Campaign


*British Journal of Cancer* (1999) **81**(4), 662–666
© 1999 Cancer Research Campaign
Article no. bjoc.1999.0744

### CA 125 regression after two completed cycles of chemotherapy: lack of prediction for long-term survival in patients with advanced ovarian cancer

**C Peters-Engl[1], A Obermair[2], H Heinzl[3], P Buxbaum[1], P Sevelda[1] and M Medl[1]**

Department of [1]Obstetrics and Gynecology, Lainz Medical Center, Wolkersbergenstraße 1, A-1130 Vienna, Austria; Departments of [2]Gynecology and Obstetrics, [3]Medical Computer Sciences/Clinical Biometrics, University Hospital of Vienna, Vienna, Austria

**Summary** The prognostic influence of CA 125 regression between the time point before surgery and after two completed courses of chemotherapy was studied in 210 patients with advanced ovarian cancer, and was compared to other well established prognostic factors. CA 125 blood samples were collected preoperatively (CA 125 pre) and 3 months after surgery (CA 125 3 mo) (at the beginning of the 3rd cycle of chemotherapy). The parameter CA 125 regression defined as $\log_{10}$ (CA 125 3 mo/CA 125 pre) was used for statistical analysis. In a survival analysis using a Cox proportional hazards model, CA 125 regression ($P$ = 0.0001), residual tumour ($P$ = 0.0001), age ($P$ = 0.0095) and grading ($P$ = 0.044) were independent variables, whereas stage of disease, histology, ascites and type of surgery failed to retain significance. Using $\log_{10}$ (CA 125 3 mo/CA 125 pre) as simple covariate in a Cox model showed a hazard ratio of 1.70 (95% confidence interval 1.32–2.19, $P$ = 0.0001). However, a detailed analysis of the interaction of time with the prognostic factor CA 125 regression on survival revealed a strong time-dependent effect with a hazard ratio of more than 6 immediately after two courses of chemotherapy, whereas within approximately 1 year the hazard ratio for the surviving patients dropped quickly to the neutral level of 1. In summary, CA 125 regression is an independent prognostic factor for survival of women with advanced ovarian cancer and allows an identification of a high-risk population among patients with advanced ovarian cancer. However, the discriminating power of serial CA 125 for long-term survival seems to be temporary and prediction of individual patients outcome is far less precise. © 1999 Cancer Research Campaign

**Keywords:** ovarian cancer; CA 125; prognosis

Patients with advanced epithelial ovarian cancer are treated with extensive cytoreductive surgery followed by several cycles of aggressive combination chemotherapy. Nevertheless, few patients stay relapse-free, and the overall prognosis, with a 5-year survival rate of 20% (Averette et al, 1996), is poor.

Survival probability is influenced by the stage of disease, residual tumour, histological type and tumour grade (Swenerton et al, 1985; Krag et al, 1989). In addition, the cancer antigen CA 125 is an established potential useful marker to monitor response to therapy and for early detection of relapse in patients with ovarian cancer (Bast et al, 1983). Several studies based on serial CA 125 measurements have addressed the above mentioned implications (Canney et al, 1984; Lavin et al, 1987; Rustin et al, 1989; Sevelda et al, 1989; Redman et al, 1990; Makar et al, 1992; Yedema et al, 1993*a*). However, the patient populations in these studies were either heterogeneous and fairly small in numbers, or the end points of outcome measurements were different (progression, response, relapse of patients in remission or survival). Despite general acceptance that CA 125 is a prognostic marker, there remains controversy as to how accurately serial CA 125 measurements can predict long-term overall survival in patients with advanced ovarian cancer.

The objective of the present study was to evaluate the prognostic influence of CA 125 regression between the time point before surgery and after two completed courses of chemotherapy on long-term survival. The rationale for the time points used was that cytoreductive surgery may cause a transient increase in CA 125, which may last for up to 2 weeks (Yedema et al, 1993*b*), and therefore prechemotherapy CA 125 sampling shortly after surgery might not be a reliable baseline parameter. In addition, the timespan between CA 125 preoperatively (CA 125 pre) and 3 months after surgery (CA 125 3 mo) reflects the effectiveness of the treatment modalities, which will both have an effect on long-term survival. The effect of the regression of serum CA 125 levels as a prognostic factor for overall survival was assessed in a Cox proportional hazards model in comparison with other established prognostic factors.

*Received 21 July 1998*
*Revised 25 March 1999*
*Accepted 12 April 1999*

*Correspondence to:* C Peters-Engl

### MATERIALS AND METHODS

Two-hundred and ten consecutive patients with International Federation of Gynaecology and Obstetrics (FIGO) stage III and IV invasive epithelial ovarian cancers and a survival of at least 3 months after surgery, were included in the present study. Non-epithelial tumours and borderline ovarian carcinomas as well as patients with secondary malignancies were excluded. All patients underwent primary surgery at the University Hospital of Vienna or the General Hospital Lainz in Vienna from 1984 to 1996. Surgery was followed by platinum-containing combination chemotherapy; subsequent courses were administered on a monthly basis. Clinical staging was performed according to the criteria established by FIGO (International Federation of Gynaecology and Obstetrics, 1987). Histological classification was based on the criteria defined

**Table 1** Patient characteristics of 210 women with advanced ovarian cancer

| | No. | % |
|---|---|---|
| Age (years) | | |
| ≤50 | 54 | 25.7 |
| >50 | 156 | 74.3 |
| FIGO stage | | |
| III | 183 | 87.1 |
| IV | 27 | 12.9 |
| Histology | | |
| Serous | 145 | 69.0 |
| Mucinous | 11 | 5.2 |
| Endometrioid | 17 | 8.1 |
| Undifferentiated | 20 | 9.6 |
| Clear-cell | 4 | 1.9 |
| Unclassified | 13 | 6.2 |
| Grade | | |
| I | 31 | 14.8 |
| II | 69 | 32.9 |
| III | 110 | 52.3 |
| Residual disease (cm) | | |
| No residual disease | 48 | 22.9 |
| <2 | 57 | 27.1 |
| 2–5 | 39 | 18.6 |
| >5 | 66 | 31.4 |
| Ascites | | |
| No ascites | 67 | 31.9 |
| ≤500 ml | 51 | 24.3 |
| >500 ml | 92 | 43.8 |
| Surgery | | |
| TAH+BSO+omentectomy | 164 | 78.1 |
| Exploratory laparotomy | 46 | 21.9 |
| CA 125 pre | | |
| < 35 U ml$^{-1}$ | 18 | 8.5 |
| <100 U ml$^{-1}$ | 41 | 19.5 |

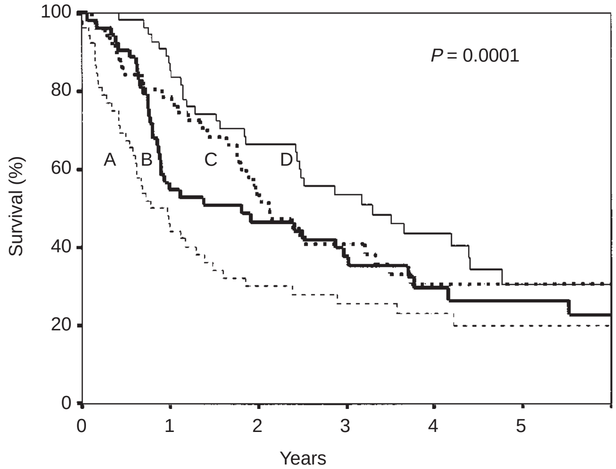

| | | | | | | | |
|---|---|---|---|---|---|---|---|
| Patients | A 52 | 23 | 14 | 11 | 7 | 5 | 3 |
| at | B 53 | 29 | 21 | 17 | 10 | 7 | 5 |
| risk: | C 51 | 39 | 26 | 17 | 11 | 7 | 1 |
| | D 54 | 46 | 33 | 23 | 18 | 8 | 5 |

**Figure 1** Kaplan–Meier plot: survival according to CA 125 regression. Curve A with >42% ($n$ = 52) or more of the initial preoperative CA 125 value, curve B: 42–14% ($n$ = 53), curve C: 14–4% ($n$ = 51) and curve D with less than 4% ($n$ = 54) of the initial CA 125 value by the World Health Organization (Serov et al, 1973). Grading was performed as outlined by Day et al (1975) Standard surgical treatment consisted of total abdominal hysterectomy (TAH), bilateral salpingo-oophorectomy (BSO) and omentectomy. Provided it was technically possible and if not contraindicated for other reason, pelvic lymphadenectomy was performed as a part of the staging procedure in those patients who had undergone cytoreductive surgery with no residual disease. Due to extensive disease some patients underwent only exploratory laparotomy. After 6 cycles of platinum-containing combination chemotherapy, the patients were followed up on a quarterly basis for the first 3 years, every 6 months for 5 years and annually thereafter. Treatment of relapsed patients included debulking surgery in few cases; otherwise polychemotherapy was used according to different study protocols. Information on follow up was obtained from medical records and the Austrian Statistical Office where all deaths in Austria are reported and registered.

Well known prognostic factors influencing survival were evaluated. Tumour stage, tumour grading and residual tumour and ascites were considered as ordered categorical variables. Histological types were categorized into three groups. Serous and unclassified histologies formed one subgroup ($n$ = 158), endometrioid, clear-cell and undifferentiated types ($n$ = 41) formed another group, and mucinous types ($n$ = 11) remained a separate entity. The patients were classified into the age groups ≤ 50 and > 50 years, and the type of surgery was classified as TAH + BSO + omentectomy versus exploratory laparotomy.

CA 125 was assayed using a radioimmunoassay (Centocor-Laboratories, Malvern, PA, USA), according to the manufacturer's instructions. CA 125 blood samples were collected preoperatively (CA 125 pre) and 3 months after surgery (CA 125 3 mo) (at the beginning of the 3rd cycle of chemotherapy), reflecting the combined effect of primary surgery and the first two courses of chemotherapy. The parameter CA 125 regression defined as $\log_{10}$ (CA 125 3 mo/CA 125 pre) was regarded as a further potential prognostic factor of survival.

### Statistical methods

Overall survival time was defined as the period between the date of CA 125 measurement 3 months after primary surgery (CA 125 3 mo) and death. Survival times of patients still alive were censored with the last follow-up date. Survival probabilities were calculated by the product limit method of Kaplan and Meier (1958). The Cox proportional hazards model (Cox, 1972) was used to assess the univariate and partial effects of prognostic factors on survival time. For ordered categorical variables a linear trend was assumed, since no deviations from linearity could be detected. Cubic spline functions with five knots were used to model the interaction of time with $\log_{10}$ (CA 125 3 mo/CA 125 pre) (Hess, 1994).

All reported $P$-values are results of two-sided tests. A $P$-value equal to or less than 5% was considered statistically significant. The SAS statistical software system (SAS Institute Inc., Cary, NC, USA) was used for calculations. For spline modelling the SAS macro of Heinzl and Kaider (1997) was used.

### RESULTS

A total of 210 patients with stage III and IV ovarian cancers were included in the study. The median age of the entire patient population was 59.7 years (range 22.1–84.0 years). At the time of analysis, 142 patients had died due to disease and 68 women were

*© 1999 Cancer Research Campaign*

**Table 2** Survival probability in patients with advanced ovarian cancer (univariate analysis)

| Prognostic factor | No. | Patients who died of disease | Kaplan–Meier 75% Quantil (years) | Univariate proportional hazard model | | |
|---|---|---|---|---|---|---|
| | | | | *P*-value | Relative risk | 95% confidence interval |
| Age | | | | 0.0001 | 2.3 | 1.48–3.4 |
| ≤ 50 | 54 | 28 | 2.38 | | | |
| > 50 | 156 | 114 | 0.72 | | | |
| FIGO stage | | | | 0.017 | 1.76 | 1.11–2.8 |
| III | 183 | 121 | 0.86 | | | |
| IV | 27 | 21 | 0.34 | | | |
| Histological type | | | | 0.37 | | |
| Serous, unclassified | 158 | 108 | 0.77 | | | |
| Mucinous | 11 | 5 | 1.11 | | 0.53 | 0.22–1.29 |
| Endometrioid, undiff., clear-cell | 41 | 29 | 0.69 | | 1.007 | 0.67–1.52 |
| Tumour grade | | | | 0.0001 | 1.66 | 1.30–2.1 |
| 1 | 31 | 11 | 3.02 | | | |
| 2 | 69 | 47 | 0.88 | | | |
| 3 | 110 | 84 | 0.65 | | | |
| Residual tumour (cm) | | | | 0.0001 | 1.74 | 1.49–2.0 |
| No | 48 | 18 | 2.40 | | | |
| < 2 | 57 | 37 | 0.93 | | | |
| 2–5 | 39 | 28 | 0.99 | | | |
| >5 | 66 | 59 | 0.46 | | | |
| Ascites | | | | 0.0002 | 1.45 | 1.19–1.76 |
| No | 67 | 35 | 1.20 | | | |
| ≤ 500 ml | 51 | 38 | 0.65 | | | |
| > 500 ml | 92 | 69 | 0.64 | | | |
| Surgery | | | | 0.0001 | 2.7 | 1.85–3.9 |
| TAH+BSO+omentectomy | 164 | 100 | 0.89 | | | |
| Exploratory laparotomy | 46 | 42 | 0.46 | | | |
| $\log_{10}$ (CA 125 3 mo/CA 125 pre) | | | | 0.0001 | see Figure 2 | see Figure 2 |
| <4% | 54 | 32 | 1.28 | | | |
| 4–14% | 51 | 33 | 1.07 | | | |
| 14–42% | 53 | 37 | 0.76 | | | |
| > 42% | 52 | 40 | 0.38 | | | |

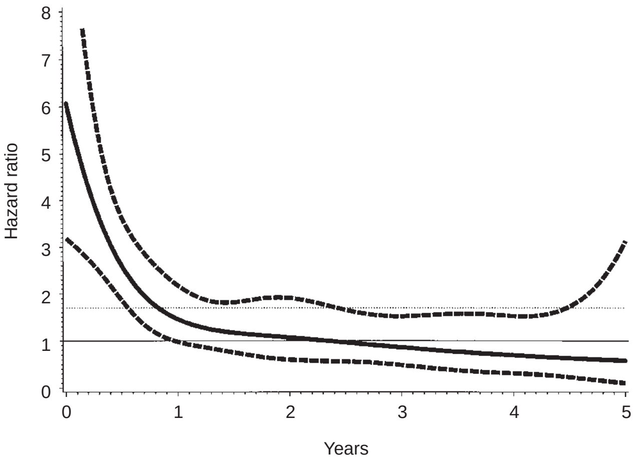

**Figure 2** Plot of hazard ratio of a unit change in $\log_{10}$ (CA 125 3 mo/CA 125 pre) versus time. The results of a time-dependent cubic spline fit (thick solid line) in a univariate Cox model are shown; 95% pointwise confidence bands (thick dashes) are added to the spline fit. Two reference lines were drawn to mark the neutral hazard ratio of 1 (thin solid line) and the result of an ordinary Cox model ignoring time-dependence (thin dotted line)

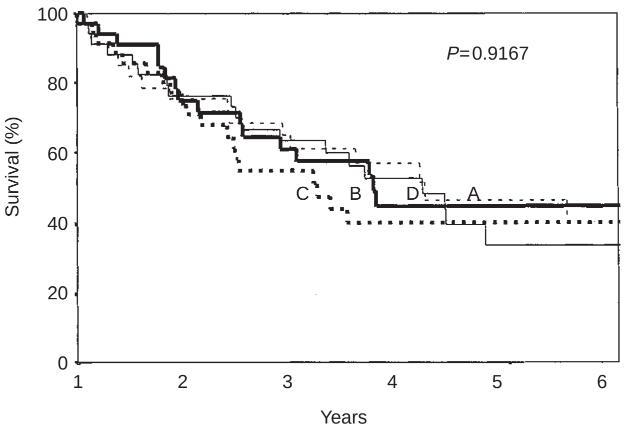

| Patients at risk: | | | | | | |
|---|---|---|---|---|---|---|
| | A 34 | 22 | 17 | 11 | 8 | 5 |
| | B 34 | 23 | 17 | 10 | 7 | 4 |
| | C 35 | 24 | 15 | 10 | 6 | 1 |
| | D 34 | 25 | 19 | 15 | 6 | 4 |

**Figure 3** Kaplan–Meier plot: Survival according to CA 125 regression of an 'exhaustion' analysis: restart the clock for the patients still alive at 1 year after the second cycle of chemotherapy and regrouping according to quartiles of the CA 125 regression. Curve A with >30% ($n$ = 34) of the initial preoperative CA 125 value, curve B: 9–30% ($n$ = 34), curve C: 3–9% ($n$ = 35) and curve D with less than 3% ($n$ = 34) of the initial CA 125 value still alive. The median overall survival interval for the study population from 3 months after surgery was 1.92 years (95% confidence interval (CI) 1.39–2.46). Surviving patients were followed up for a median of 4.73 years (range 0.93–12.22 years) estimated from 3 months after surgery. Detailed patient characteristics are summarized in Table 1. Overall, the median preoperative CA 125 value was 453.2 U $ml^{-1}$ (range 14.0–19 122.0 U $ml^{-1}$, interquartile range 160.7–913.2 U $ml^{-1}$) versus 30.6 U $ml^{-1}$ (range 1.0–

© 1999 Cancer Research Campaign

**Table 3** Results of a multiple proportional hazards model (Cox model)

| Prognostic factor | *P*-value | Relative risk | 95% Confidence interval |
|---|---|---|---|
| Age | 0.0095 | 1.83 | 1.16–2.9 |
| FIGO stage | 0.40 | 0.81 | 0.49–1.33 |
| Histological type | 0.33 | | |
| Serous + unclassified vs mucinous | | 0.52 | 0.21–1.29 |
| Serous + unclassified vs endom. + undiff. + clear | | 1.07 | 0.70–1.65 |
| Tumour grade | 0.044 | 1.31 | 1.008–1.7 |
| Residual tumour | 0.0001 | 1.59 | 1.28–1.98 |
| Ascites | 0.32 | 1.12 | 0.90–1.41 |
| Surgery | 0.89 | 0.97 | 0.62–1.53 |
| $Log_{10}$ (CA 125 3 mo/CA 125 pre) | 0.0001 | data not shown | data not shown |

8022.0 U $ml^{-1}$, interquartile range 16.0 U–158.3 U $ml^{-1}$) after surgery and two subsequent completed cycles of platinum containing chemotherapy respectively.

For descriptive reasons the regression of CA 125 was categorized into four groups, each with a nearly equal number of patients: Group A with more than 42% ($n$ = 52) of the initial preoperative CA 125 value after 3 months, Group B: 14–42% ($n$ = 53), Group C: 4–14% ($n$ = 51) and group D with less than 4% ($n$ = 54) of the initial CA 125 value. Survival probabilities according to Kaplan–Meier are shown in Figure 1.

For the Cox proportional hazards model the regression of CA 125 was defined as $log_{10}$ (CA 125 3 mo/CA 125 pre). Univariate analysis identified a number of significant prognostic factors for survival. The impact of each of the evaluated prognostic factors is shown in Table 2. Using $log_{10}$ (CA 125 3 mo/CA 125 pre) as simple covariate in a univariate Cox model revealed a hazard ratio of 1.70 (95% CI, 1.32–2.19, $P$ = 0.0001). A more refined analysis (Hess, 1994) shows that this may be a misleading result. Actually the effect of $log_{10}$ (CA 125 3 mo/CA 125 per) on survival is strongly time-dependent ($P$ = 0.0001, Figure 2). Immediately after two courses of chemotherapy a hazard ratio of more than 6 (95% confidence bands shown in Figure 2) was estimated from the data. Within approximately 1 year, the hazard ratio drops quickly to the neutral level of 1. These results show that the change in CA 125 3 months after surgery compared to presurgery is an important prognostic factor. However, its discriminating power is only temporary. In the first year there is a clear distinction between the groups, whereas afterwards all surviving patients stay on a common risk level. Inspecting the Kaplan–Meier survival plot of an ‘exhaustion’ analysis by starting 12 months into the follow-up puts emphasis on this finding (Figure 3).

Finally, all prognostic variables were entered as covariates in a Cox proportional hazards model (Table 3). Of the variables which showed a significant influence on survival in the univariate analysis, only CA 125 regression, residual tumour, age and grading remained as independent variables, whereas stage of disease, histology, ascites and type of surgery failed to retain significance. Patients above 50 years of age had a 1.83-fold higher risk (95% CI 1.16–2.9) of dying from ovarian cancer, while an increase in tumour grading resulted in a 1.31-fold higher risk (95% CI 1.008–1.7). The increasing amount of residual tumour correlated significantly with survival (RR 1.59; 95% CI 1.28–1.98). Mucinous tumours showed a non-significant better prognosis (RR 0.52). $Log_{10}$ (CA 125 3 mo/CA 125 pre) shows the same strong time-dependent effect on survival in the multiple Cox model ($P$ = 0.0001; data not shown) as in the univariate Cox model (Figure 2).

### DISCUSSION

Aim of this study was to investigate how accurately serial serum CA 125 measurement can predict long-term survival of patients with advanced ovarian cancer. This study is one of the largest series addressing this question with complete data of 210 patients available for analysis. In the present study CA 125 serum level regression was, as well as residual tumour, age and tumour grade an additional independent prognostic factor for survival in patients with stage III and IV ovarian cancer. Our results show that CA 125 regression from preoperative levels to 3 months after surgery (including two cycles of chemotherapy) can provide valuable prognostic information concerning overall survival; however, the discriminating power of CA 125 for survival seems to be restricted to the first 12 months.

The phenomenon of CA 125 regression and response to initial chemotherapy has been well researched and documented by several authors (Bast et al, 1983; Canney et al, 1984; Lavin et al, 1987; Sevelda et al, 1989; Redman et al, 1990; Mogenson, 1992). The effect of CA 125 regression is not only important to judge the patient’s response to cytotoxic chemotherapy but also allows a distinction between long-term and short-term survivors. In order to improve the distinction between short-term and long-term survivors, the patients were divided into four groups according to their CA 125 regression levels. All groups comprised equal numbers of patients. Women with a CA 125 regression to less than 4% of the initial baseline value had a median survival of 3.29 years. This should be compared with a median survival of 2.11 years for CA 125 regression between 4% and 14%, and 1.80 years for CA 125 regression between 14% and 42% respectively. Survival of patients with tumour marker regression to more than 42% of the baseline values did worst, with a median survival of 0.87 years. The results presented are consistent with findings obtained by earlier studies on serial CA 125 measurement (Rustin et al, 1989; Hawkins et al, 1989; Hunter et al, 1990; Fayers et al, 1993; Yedema et al, 1993).

These results may indicate that there might be no point in continuing cytotoxic chemotherapy in this subset of patients with a poor long-term prognosis; it might be better to offer them less toxic, palliative treatment or to randomize them into studies with

© 1999 Cancer Research Campaign 

novel, potentially curative drugs, as suggested by other authors (Rustin et al, 1989; Sevelda et al, 1989; Mogenson, 1991).

However, this conclusion has to be interpreted with great caution, because an additional new aspect in the serial CA 125 measurement of advanced ovarian cancer patients was found in our study when we assessed the interaction of tumour marker regression with time. Our results show that patients surviving the critical interval of approximately 1 year will have the same long-term prognosis that they would have had if they had had a greater CA 125 fall. A similar conclusion was made by Fayers et al (1993) showing nearly 20% of the patients in the bad prognostic group to be alive and progression-free at 12 months. This is in accordance with our results and further supports our findings.

Nevertheless, two major biases might have effected the interpretation of our data: first, two different phases of CA 125 fall were included in our measurements, the effect of surgical debulking and the initial response to chemotherapy. However, the data presented were highly significant in a multivariate survival analysis including residual disease as marker for effect of surgery and tumour reduction. Moreover, the half-life of CA 125 was shown to be independent on the tumour size at the start of chemotherapy (Van der Burgh et al, 1988). Secondly, in spite of a good response, patients with an initially low CA 125 will have a low fall in CA 125 and might be classified in the same group as patients with a high pre CA 125 and low response which is a general problem when analysing dynamic processes. We were aware of this fact and in order to overcome this problem subgroup analysis including only patients with CA 125 pre > 35 U $ml^{-1}$ and CA 125 pre > 100 U $ml^{-1}$ was performed. Analysis revealed a similar time-dependent effect of CA 125 regression, confirming the interpretation of our data (results not shown).

In conclusion, CA 125 regression was an independent prognostic factor for survival in women with advanced ovarian cancer, and this effect is limited to the duration of 1 year after surgery. After approximately 1 year, CA 125 did not add information to the long-term prognosis of these patients. The discriminating power solely on the basis of serial CA 125 tumor marker levels seems to be temporary and prediction of individual patients long-term outcome is not accurate enough.

### ACKNOWLEDGEMENTS

This study was in part supported by the Ludwig Boltzmann Institute for Gynecologic Oncology and Reproductive Medicine, Vienna, Austria.

### REFERENCES

Atack DB, Nisker JA, Allen HH, Tustanoff ER and Levin L (1986) CA 125 surveillance and second-look laparotomy in ovarian carcinoma. *Am J Obstet Gynecol* **154**: 287–289

Averette HE, Janicek MF and Menck HR (1996) The National Cancer Data Base report on ovarian cancer. American College of Surgeons Commission on Cancer and the American Cancer Society. *Cancer* **76**: 1096–1103

Bast RC Jr, Klug TL, St John E, Jenison E, Niloff JM, Lazarus H, Berkowitz RS, Laevitt T, Griffiths CT, Parker L, Zurawski VR Jr and Knapp RC (1983) A radioimmunoassay using a monoclonal antibody to monitor the course of epithelial ovarian cancer. *N Engl J Med* **309**: 883–887

Canney PA, Moore M, Wilkinson PM and James RD (1984) Ovarian cancer antigen CA-125: a prospective clinical assessment of its role as a tumour marker. *Br J Cancer* **50**: 765–769

Cox DR (1972) Regression models and life tables (with discussion). *J Royal Statist Soc* **B 34**: 187–220

Day TG, Gallagher HS and Rudledge FN (1975) Epithelial carcinoma of the ovary: prognostic importance of histologic grade. *J Natl Cancer Inst* **42**: 15–21

Fayers PM, Rustin G, Wood R, Nelstrop A, Leonard RCF, Wilkinson P, Cruickshank D, McAllister EJ, Redman CWE, Parker D, Scott IV, Slevin ML and Roulston JE (1993) The prognostic value of serum CA 125 in patients with advanced ovarian carcinoma: an analysis of 573 patients by the Medical Research Council Working Party on Gynaecological Cancer. *Int J Gynecol Cancer* **3**: 285–292

Hawkins RE, Roberts K, Wiltshaw E, Mundy J, Fryatt IJ and McCready VR (1989) The prognostic significance of the half-life of serum CA 125 in patients responding to chemotherapy for epithelial ovarian carcinoma. *Br J Obstet Gynaecol* **96**: 1395–1399

Heinzl H and Kaider A (1997) Gaining more flexibility in Cox proportional hazards regression models with cubic spline functions. *Comp Meth Prog Biomed* **54**: 201–208

Hess KR (1994) Assessing time-by-covariate interactions in proportional hazards regression models using cubic spline functions. *Statist Med* **13**: 1045–1062

Hunter VJ, Daly L, Helms M, Soper JT, Berchuck A, Clarke-Pearson DL and Bast RC (1990) The prognostic significance of CA 125 half-life in patients with ovarian cancer who received primary chemotherapy after surgical cytoreduction. *Am J Obstet Gynecol* **163**: 1164–1167

International Federation of Gynaecology and Obstetrics (1987) Changes in definitions of clinical staging for carcinoma of the cervix and ovary. *Am J Obstet Gynecol* **156**: 263–264

Kaplan EL and Meier P (1958) Nonparametric estimation from incomplete observations. *J Am Statist Assoc* **53**: 457–481

Krag KJ, Canellos GP, Griffiths CT, Knapp RC, Parker LM, Welch WR, Klatt M and Andersen J (1989) Predictive factors for long term survival in patients with advanced ovarian cancer. *Gynecol Oncol* **34**: 88–93

Lavin PT, Knapp RC, Malkasian G, Whitney CW and Berek JC (1987) CA-125 for the monitoring of ovarian carcinoma during primary therapy. *Obstet Gynecol* **69**: 223–227

Makar AP, Kristensen GB, Børmer OP and Claes GT (1993) Serum CA 125 level allows early identification of non-responders during induction chemotherapy. *Gynecol Oncol* **49**: 73–79

Mogenson O, Mogenson B and Jakobsen A (1990) Predictive value of CA 125 during early chemotherapy of advanced ovarian cancer. *Gynecol Oncol* **37**: 44–46

Mogensen O (1992) Prognostic value of CA 125 in advanced ovarian cancer. *Gynecol Oncol* **44**: 207–212

Redman CWE, Blackledge Jr, Kelly K, Powell J, Buxton EJ and Luesly DM (1990) Early serum CA 125 response and outcome in epithelial ovarian cancer. *Eur J Cancer* **26**: 593–596

Rustin GJS, Gennings JN, Nelstrop AE, Covarrubias H, Lambert HE and Bagshawe KD (1989) Use of CA-125 to predict survival of patients with ovarian carcinoma. *J Clin Oncol* **11**: 1667–1671

Serov SF, Scully RE and Sorbin LH (1973) Histological typing of ovarian tumors. In: *International Histological Classification of Tumors*, pp. 17–18. World Health Organization: Geneva, Switzerland

Sevelda P, Schemper M and Spona J (1989) CA 125 as an independent prognostic factor for survival in patients with epithelial ovarian cancer. *Am J Obstet Gynecol* **161**: 1213–1216

Swenerton KD, Hislop TG, Spinelli J, LeRiche JC, Yang N and Boyes DA (1985) Ovarian carcinoma: a multivariate analysis of prognostic factors. *Obstet Gynecol* **65**: 264–269

Van der Burgh MEL, Lammes FB, Van Putten WJL and Stotter G (1988) Ovarian cancer: the prognostic value of the serum half-life of CA-125 during the induction of chemotherapy. *Gynecol Oncol* **30**: 307–312

Yedema CA, Kenemans P, Voorhorst F, Bon G, Schijf C, Beex L, Verstraeten A, Hilgers J and Vermorken J (1993*a*) CA 125 half-life in ovarian cancer: a multivariate survival analysis. *Br J Cancer* **67**: 1361–1367

Yedema CA, Kenemans P, Thomas CMG, Massuger LFAG, Wobbes TH, Verstraeten AA, van Kamp GJ, Hilgers J (1993*b*) CA 125 serum levels in the early postoperative period do not reflect the outcome of cytoreductive surgery. *Eur J Cancer* **29**: 966–971

 © 1999 Cancer Research Campaign
